# Propofol-based deep sedation for colonoscopy: does sufentanil, alfentanil or ketamine help? A propensity score weighted retrospective study

**DOI:** 10.7717/peerj.19146

**Published:** 2025-03-10

**Authors:** Michele Fostier, Quentin Delhez, Gintare Januleviciute, Laurent Bairy

**Affiliations:** Anesthesia Department, CHU Ucl Namur, Yvoir, Namur, Belgium

**Keywords:** Anesthesia, Sedation, Endoscopy, Propofol

## Abstract

**Introduction:**

Gastrointestinal endoscopy is a frequent procedure for diagnosing and following up on various digestive disorders. It is often conducted under propofol sedation. The aim of this work is, first, to determine whether the addition of sufentanil, alfentanil, or ketamine to propofol has a propofol-sparing effect and, secondarily, how these drugs affect the patients’ hemodynamic parameters and oxygenation as well as the duration of the procedure.

**Methods:**

Data from patients who underwent colonoscopy were extracted from the anesthesia records database and divided into four groups. Patients received either propofol or a combination of propofol and sufentanil, alfentanil, or ketamine. After inverse propensity weighting, we determined the average treatment effects for each group for the primary and secondary endpoints.

**Results:**

Sufentanil was associated with a less than 10% decrease in propofol consumption. Alfentanil and ketamine showed no propofol-sparing effect. Sufentanil was associated with 2 min shorter procedures. Alfentanil was associated with more patients presenting hypoxemia and had no propofol-sparing effect.

**Conclusion:**

Should a balanced sedation technique be chosen, sufentanil appears to be the adjuvant of choice, given its propofol-sparing effect and the absence of induced hypoxemia. A further prospective study is necessary to explain the lack of propofol-sparing effect of ketamine and alfentanil and confirm and explain the negative impact of alfentanil on patients’ oxygenation.

## Introduction

Colonoscopy is a frequent procedure that causes anxiety and discomfort in patients. It has been carried out under sedation more frequently as the years passed to relieve patients from these unwanted effects. Various cultural and local factors influence the way this sedation is conducted, such as local regulations and guidelines, the physician’s ability to use certain drugs, or costs induced by the intervention of an anesthesiologist (Rex & Khalfan, 2005). There are several benefits to performing a colonoscopy under sedation. In addition to better patient comfort, it also improves the endoscopist comfort (Lee et al., 2011; Akarsu Ayazoglu & Uzman, 2021) and increases the lesion detection rate (Zhou et al., 2021a). As the patient’s medical situation requires it, a gastroscopy, which also causes some discomfort, may also be performed during the same sedation.

The most used medications for these sedations are midazolam, fentanyl, sufentanil, and propofol, although a wide variety of drugs have been used for this purpose. Gastroenterologists often use midazolam either with or without opioids, as propofol administered by a non-anesthesiologist is a controverted matter and not permitted in every jurisdiction (De Cosmo, Levantesi & Del Vicario, 2020). The literature is scarce about the drugs used by anesthesiologists to sedate patients for digestive endoscopy, but propofol seems to be the most used drug in this scenario (Zhou et al., 2021b). Except in the United States, where this mode has not received regulatory approval, the prevalent administration mode of propofol is *via* a target-controlled infusion. One side effect of propofol is the pain experienced by some patients when being injected. This pain can be attenuated with an intravenous (IV) bolus of lidocaine, explaining why propofol and lidocaine are a customary mix used by anesthesiologists. In the case of balanced sedation, anesthesiologists can add adjuvants to reduce the propofol dose and adverse effects (Brown, Pavone & Naranjo, 2018). This retrospective study aims to determine whether the use of sufentanil, alfentanil, or ketamine has a propofol-sparing effect and, second, whether it affects the duration of the procedure or the respiratory and hemodynamic parameters of the patient.

## Materials and Methods

This study was approved by the local ethics commitee of CHU UcL Namur (“Comité d’éthique médicale du CHU UcL Namur—site Godinne”, approval ref. 125/2021). Patient consent was not required for this work, as this study used data from medical records without identifying data. This waiver of patient consent was approved by the institutional ethics committee per the Belgian legislation. Furthermore, this study was conducted per the Declaration of Helsinki and the European General Data Protection Regulation (GPDR).

We reviewed the electronic anesthesia records database, and data of all patients who underwent a colonoscopy, with or without gastroscopy, in the day surgery center of a tertiary university hospital in the period from January 2017 to July 2021 were extracted and divided into four groups:

- The P group in which patients received only a target-controlled infusion of propofol and an intravenous (IV) bolus of lidocaine 0.5–1 mg/kg.
- The S group included patients who received a target-controlled infusion of propofol, a 0.5–1 mg/kg lidocaine IV bolus, and an additional 5 mcg IV bolus of sufentanil.
- The A group in which they received a target-controlled infusion of propofol, a 0.5–1 mg/kg lidocaine IV bolus, and a 0.5 mg IV bolus of alfentanil.
- The K group in which patients received a target-controlled infusion of propofol, a 0.5–1 mg/kg lidocaine IV bolus, and 25–50 mg of ketamine IV.

All patients received supplemental oxygen at 2 to 6 L/min *via* a facemask or nasal cannula.

The same team of five endoscopists performed all endoscopic procedures.

We performed an inverse propensity weighting (IPTW) analysis to reduce group inhomogeneity and maximize available data for analysis. Age, sex, American Society of Anesthesiologists (ASA) class, gastroscopy, and body mass index (BMI) were used to compute the propensity score using a three-layer neural network (24/12/4). We used standardized mean difference (SMD) to assess the balance of baseline characteristics. An SMD less than 0.1 was considered a low imbalance. Weights were calculated for the average treatment effect (ATE) and subsequently stabilized. Linear regressions were used to determine the ATE for the primary outcome (propofol dose administered) and the secondary outcomes (hypoxemia, bradycardia, hypotension, tachycardia, hypertension, procedure duration, and length of stay in the outpatient center) for each group. Hypoxemia, hypotension, hypertension, bradycardia, and tachycardia were defined, respectively, as an occurrence of SpO2 <= 90%, mean arterial pressure (MAP) < 55 mmHg, MAP > 105 mmHg, heart rate (HR) < 50, and HR > 120 bpm. S, A, and K groups were compared to the P group and not with each other. Patients with incomplete datasets were excluded. We did not correct for multiple analyses as secondary outcomes were considered exploratory. Secondary outcomes are described by their effect estimate and 95% confidence interval (95% CI).

Statistical analyses were performed using R software version 4.3. The neural network and weight determination were coded in Python 3.11 with the Tensorflow and Keras frameworks. A *p* < 0.05 was considered statistically significant.

## Results

A total of 5,333 patients underwent colonoscopy with or without an associated gastroscopy during the study period. Of these patients, 2,227 patients were excluded because of incomplete records. The remaining 3,106 patients received a target-controlled propofol infusion and an intravenous bolus of lidocaine 0.5–1 mg/kg. Of these patients, 2,648 received only propofol, 311 received 5–10 mcg sufentanil, lidocaine, and propofol, 83 received 0.5 mg alfentanil, lidocaine, and propofol, and 64 received 10–50 mg ketamine, lidocaine, and propofol.

There was a significant imbalance in the A group as three times more patients underwent a gastroscopy, a known cause of oxygenation problems (Long et al., 2012; Nay et al., 2021). This imbalance disappeared after IPTW. Table 1 shows patients’ characteristics before and after the weighting procedure.

**Table 1:** Population description (count (percentage) or mean (SD)). OSAS, obstructive sleep apnea syndrome; ICM, ischemic cardiomyopathy; COPD, chronic obstructive pulmonary disease.

	Before weighting					After weighting				
	P	S	A	K	SMD	P	S	A	K	SMD
*n*	2,648	311	83	64		2,648.9	307.4	74	59.1	
OSAS (%)	133.0 (5.2)	18.0 (6.0)	2.0 (2.5)	1.0 (1.6)	0.139	134.8 (5.3)	12.0 (4.0)	2.2 (3.1)	1.2 (2.2)	0.091
Hypertension (%)	915.0 (36.1)	107.0 (35.4)	32.0 (40.5)	20.0 (32.8)	0.083	910.7 (35.9)	104.0 (35.1)	38.0 (54.0)	18.4 (32.3)	0.226
ICM (%)	159.0 (6.3)	16.0 (5.3)	1.0 (1.3)	2.0 (3.3)	0.152	158.4 (6.2)	16.4 (5.5)	0.4 (0.6)	1.7 (3.0)	0.183
COPD (%)	396.0 (15.6)	43.0 (14.2)	15.0 (19.0)	5.0 (8.2)	0.166	396.2 (15.6)	46.1 (15.5)	14.5 (20.6)	3.5 (6.2)	0.219
Mallampati (%)	243 missing	23 missing	8 missing	11 missing	0.317					0.317
1	1,071.0 (44.5)	105.0 (36.5)	35.0 (46.7)	30.0 (56.6)		1,069.0 (44.4)	108.9 (38.6)	25.8 (39.1)	23.8 (50.6)	
2	907.0 (37.7)	121.0 (42.0)	24.0 (32.0)	17.0 (32.1)		899.8 (37.4)	125.0 (44.4)	27.1 (41.0)	15.5 (33.0)	
3	366.0 (15.2)	54.0 (18.8)	12.0 (16.0)	3.0 (5.7)		375.9 (15.6)	42.0 (14.9)	12.1 (18.3)	2.9 (6.3)	
4	61.0 (2.5)	8.0 (2.8)	4.0 (5.3)	3.0 (5.7)		62.8 (2.6)	5.9 (2.1)	1.0 (1.6)	4.8 (10.1)	
Smoking (%)	422.0 (17.6)	65.0 (23.2)	15.0 (19.5)	8.0 (14.0)	0.127	424.4 (17.7)	64.1 (23.3)	15.5 (22.3)	6.0 (11.2)	0.182
Age (mean(SD))	59.46 (14.95)	58.46 (15.17)	56.20 (14.69)	55.69 (17.25)	0.146	59.19 (15.01)	59.15 (14.85)	57.75 (12.01)	56.26 (18.74)	0.108
Asa (%)					0.156					0.24
1	156.0 (5.9)	16.0 (5.1)	5.0 (6.0)	4.0 (6.2)		154.5 (5.8)	15.3 (5.0)	8.7 (11.8)	8.1 (13.6)	
2	2,332.0 (88.1)	275.0 (88.4)	74.0 (89.2)	54.0 (84.4)		2,332.2 (88.0)	277.3 (90.2)	62.0 (83.8)	47.4 (80.1)	
3	150.0 (5.7)	19.0 (6.1)	3.0 (3.6)	6.0 (9.4)		152.2 (5.7)	14.1 (4.6)	2.4 (3.2)	3.7 (6.3)	
4	10.0 (0.4)	1.0 (0.3)	1.0 (1.2)	0.0 (0.0)		10.0 (0.4)	0.8 (0.2)	0.9 (1.2)	0.0 (0.0)	
Gastroscopy (%)	870.0 (32.9)	89.0 (28.6)	66.0 (79.5)	20.0 (31.2)	0.591	901.9 (34.0)	98.6 (32.1)	25.1 (34.0)	18.5 (31.3)	0.036
Bmi (mean(SD))	26.73 (9.37)	27.22 (10.79)	27.81 (4.56)	27.99 (6.30)	0.09	26.74 (9.16)	27.88 (15.20)	27.91 (4.46)	26.82 (5.87)	0.094
Sex = F (%)	1,337.0 (50.5)	164.0 (52.7)	39.0 (47.0)	29.0 (45.3)	0.086	1,340.5 (50.6)	154.1 (50.1)	34.4 (46.5)	30.6 (51.7)	0.054
Biopsy (%)	1,226.0 (46.4)	147.0 (47.4)	54.0 (65.1)	16.0 (25.0)	0.43	1,228.5 (46.5)	146.4 (47.8)	46.9 (63.3)	13.3 (22.5)	0.443
Polypecctomy (%)	390.0 (14.8)	57.0 (18.4)	9.0 (10.8)	8.0 (12.5)	0.119	389.2 (14.7)	56.8 (18.5)	11.1 (14.9)	7.0 (11.9)	0.094
Mallampati>2 (%)	427.0 (17.8)	62.0 (21.5)	16.0 (21.3)	6.0 (11.3)	0.154	438.6 (18.2)	47.9 (17.0)	13.1 (19.9)	7.7 (16.4)	0.051

There was no failed endoscopy because of a problem due to sedation. All unfinished procedures were because of insufficient preparation. The gastroenterologist’s patient tolerance report was either not present and thus considered good, or the tolerance report was left to the anesthesiologist.

Table 2 shows the outcomes for each group. The propofol amount is statistically different across the groups, with a lower amount in the S group (−33 mg compared to the P group). There is a trend for the patients in the A group to receive more propofol, although this is not statistically solid evidence.

**Table 2:** Primary and secondary endpoints.

	Group			
	P	S	A	K
Propofol (mg)	475.2 (466.1–484.4)	438.6 (416.6–460.5) *p* = 0.003	624.2 (465.6–782.8) *p* = 0.066	453.4 (390.2–516.6) *p* = 0.503
Duration (min)	33 (32–33)	31 (29–32)	36 (31–41)	35 (32–40)
Length of stay (hr)	2.38 (2.35–2.42)	2.36 (2.26–2.46)	2.28 (2.10–2.46)	2.39 (2.10–2.69)
Hypoxemia (OR)	0.08 (0.07–0.09)	1.15 (0.73–1.82)	3.66 (1.38–9.69)	1.29 (0.55–3.00)
Bradycardia (OR)	0.11 (0.10–0.12)	1.00 (0.67–1.49)	0.38 (0.17–0.83)	0.47 (0.13–1.66)
Hypotension (OR)	0.41 (0.38–0.45)	1.25 (0.96–1.62)	1.17 (0.56–2.47)	1.37 (0.76–2.48)
Tachycardia (OR)	0.05 (0.05–0.06)	0.89 (0.51–1.53)	2.83 (0.83–9.61)	2.27 (0.89–5.80)
Hypertension (OR)	0.33 (0.30–0.36)	0.75 (0.55–1.01)	1.06 (0.45–2.54)	1.41 (0.77–2.58)

**Note:**

OR: odds ratio, (95% confidence interval).

The average duration of a colonoscopy, including sedation, is 33 min in the P group and 2 min shorter (95% CI [29–32]) in the S group. The differences seen in the A and K groups are not different from those in the P group. There is no difference in the length of stay in the daycare center.

Hypoxemia, defined as SpO2 <= 90%, occurs in 8.2% of the patients in the P group. The odds ratio for desaturation for the A group is 3.66 (95% CI [1.38–9.69]).

The incidence of bradycardia, defined as the occurrence of a heart rate lower than 40 bpm, in the P group is 9.9%. The OR of the A group is 0.38 (95% CI [0.17–0.83]), showing less frequent bradycardia in this group.

The odds ratio of tachycardia, hypertension, and hypotension are not different from the P group.

## Discussion

Procedural sedation for colonoscopies is a much-debated subject. Drugs used to sedate the patients are chosen according to local regulations, the background of the person who provides the sedation, and the availability of the drugs (Rex & Khalfan, 2005; Childers, Williams & Sonnenberg, 2015). In 2021, Zhou et al. (2021b) reported that propofol is the preferred drug for anesthesiologists to sedate patients undergoing a colonoscopy. Most of these patients also received an opioid. The explanation for this mixture is that colonoscopy is a painful procedure, and providing a drug that relieves pain could lead to a diminished dose of propofol administered to the patient, ultimately decreasing the likelihood of side effects of both drugs. This refers to balanced anesthesia (Brown, Pavone & Naranjo, 2018). In this work, a propofol-sparing effect is only seen with sufentanil, allowing a less than 10% reduction of propofol. In contrast, neither alfentanil nor ketamine showed a decrease in injected propofol. Patients who received alfentanil even showed a trend toward greater propofol consumption. The explanation could be the shorter analgesia provided by alfentanil. Its effect stops before the procedure ends, leading the clinician to administer more propofol to maintain adequate sedation. This pattern was already seen in the work of Türk et al. (2013), where they compared alfentanil with fentanyl for colonoscopies. Patients who received alfentanil also received more propofol than those who received fentanyl. Alfentanil and sufentanil are both fentanyl derivatives with, respectively, a short and mid-acting period. After a single IV bolus, alfentanil has a lower onset (0.75 *vs*. 1 min for sufentanil), and the end of its clinical effect is determined by the redistribution half-life of respectively, 4–17 min and 15–20 min, explaining the differences of the duration of peak clinical effect of 15 and 30 min between both drugs (Ziesenitz et al., 2018). Alfentanil also has a 20 times lower therapeutic index than sufentanil (Kumar, 2022). This leads to alfentanil having a higher propensity to induce apnea, as already described in previous papers (Hull & Jacobson, 1983; Miner et al., 2009), which explains the three times more frequent desaturation events seen in the A group. This effect was also seen in a previous publication where Nilsson et al. (2012) reported more respiratory events in patients who had received alfentanil when compared to propofol alone.

Miner et al. (2009) also concluded that alfentanil has no benefit for procedural sedation in the emergency department and that more patients need airway maneuvers to avoid hypoxemia. In contrast, these results were not confirmed by others who did not report such adverse effects on oxygenation (Lei, Zhang & Huang, 2024), where they concluded that alfentanil is safer than sufentanil. Nevertheless, these procedures did not involve the airway, as does gastroscopy, and patients received a higher dose of sufentanil.

Ketamine does not provide any propofol-sparing effect or benefit on hemodynamic and respiratory parameters, which contradicts previous literature (David & Shipp, 2011; Yin et al., 2019; Eberl et al., 2020). The equivalent dose of ketamine studied in these works was in line with what the patients received in this work (median: 0.4 mg/kg [0.43–0.94]). We do not have a clear explanation for this absence of effect, but the K group is relatively small. Future prospective work must confirm and explain this absence of beneficial effects.

Regarding the procedure duration, only the patients receiving sufentanil had a slightly shorter procedure (−2 min). A deeper and more stable level of sedation may explain this. Zuber-Jerger & Kullmann (2006) found that the quality of sedation was linked to the time to caecal intubation, although this relationship is disputed. This shorter time is not seen in the other two groups. The length of stay in the daycare center is not affected by the drugs used for sedation. This is undoubtedly due to the short-acting medications used in this work.

Regarding the dosage of the drugs used in this work, there is no unanimous dosage for sufentanil, alfentanil, or ketamine for procedural sedation. For alfentanil, 10 mcg/kg seems to be a commonly used dose (Türk et al., 2013), and the median dose received by the group A patient is 12 mcg/kg (interquartile range (IQR): 11–14 mcg/kg). For sufentanil, a dose of 0.1 mcg/kg is often used (Yin et al., 2019), the median dose of group S was 0.6 mcg/kg (IQR: 0.04–0.07 mcg/kg), and patients receiving ketofol, a 1:1 mix of ketamine and propofol, often receive 0.5–1 mg/kg ketamine for procedural sedation. (Yin et al., 2019; David & Shipp, 2011) The patients from group K received 0.4 mg/kg (IQR: 0.3–0.5 mg/kg) ketamine. The dosage used in this work seems to align with the literature about procedural sedation except for sufentanil, as patients receive nearly half the 0.1 mcg/kg dose.

### Limitations

The statistical treatment applied here, consisting of weighting patients according to their propensity score, should overcome the shortcomings of the retrospective design of this study by balancing covariates across groups and maximizing data available for analysis. However, a covariate influencing the choice of an adjuvant not considered in this study is possible but unlikely. Another potential criticism could be that the level of sedation was not assessed. It is never done in clinical practice. We believe that the ideal depth of sedation for colonoscopies is when the patient does not move and, therefore, does not interfere with the performance of the examination.

One could also argue about the relatively constant dose of adjuvant administered to the patients, which reflects the daily practice and is seen in others’ work (Nay et al., 2021).

Further prospective work must be done to confirm and try to explain the lack of propofol-sparing effect of ketamine and alfentanil in the setting of gastrointestinal endoscopy and the negative effect of alfentanil on hypoxemia.

## Conclusions

We do not demonstrate a clear benefit of using an adjuvant compared to a more straightforward propofol regimen. The injection of a 5 mcg sufentanil bolus has a slight propofol-sparing effect and seems to shorten the procedure. On the other hand, alfentanil induces more hypoxemia (OR = 3.66) than propofol alone and does not have a propofol-sparing effect.

Based on this retrospective study, we advise against the use of alfentanil, which has a too-short duration of action, therefore inducing an unstable level of sedation. Future prospective work needs to confirm this. Sedation using propofol alone remains the safest technique.

Should a balanced sedation technique be chosen, sufentanil appears to be the adjuvant of choice, given its propofol-sparing effect and the absence of induced hypoxemia. A prospective study would help clarify the lack of ketamine’s effect.

## Supplemental Information

10.7717/peerj.19146/supp-1 Supplemental Information 1 Anonymised patient data.

10.7717/peerj.19146/supp-2 Supplemental Information 2 Codebook.

## Additional Information and Declarations

### Data Availability

The following information was supplied regarding data availability:

Raw data is available as a Supplemental File.
